# Decreased IL-27 Negatively Correlated with Th17 Cells in Non-Small-Cell Lung Cancer Patients

**DOI:** 10.1155/2015/802939

**Published:** 2015-04-19

**Authors:** Minchao Duan, Zhengqing Ning, Zhijun Fu, Jianquan Zhang, Guangnan Liu, Qiu Wei, Xiaoyu Zheng

**Affiliations:** ^1^Department of Respiratory Medicine, The Eighth People's Hospital of Nanning, Nanning, Guangxi 530001, China; ^2^Department of Respiratory Medicine, The First People's Hospital of Nanning, Nanning, Guangxi 530001, China; ^3^Department of Respiratory Medicine, The First Affiliated Hospital, Guangxi Medical University, Nanning, Guangxi 530021, China

## Abstract

The presence of Th17 cells and IL-27 is observed in a variety of inflammatory associated cancers. However, there are some data on the role of Th17 cells and IL-27 in the regulation of immune reactions in non-small-cell lung cancer (NSCLC). The aim of this study is to assess the variation of Th17 cells and IL-27 in the peripheral blood (PB) of patients with NSCLC. The proportion of Th17 cells in peripheral blood mononuclear cells (PBMCs) was evaluated by flow cytometry. The serum concentrations of IL-27 and IL-17 were measured by enzyme-linked immunosorbent assay (ELISA). The mRNA expression of ROR*γ*t and IL-27 in the peripheral blood was examined by real-time quantitative polymerase chain reaction (QPCR). Expression of IL-27 was lower in NSCLC patients compared with normal controls. The frequency of Th17 cells was increased in NSCLC patients, accompanied by the upregulation of IL-17 and ROR*γ*t. IL-27 negatively correlated with the number of Th17 cells and the ROR*γ*t mRNA. Our results indicate that IL-27 might inhibit Th17 differentiation in NSCLC patients and better understanding of the regulatory effects of IL-27 on Th17 cells may shed light on potential new targets in cancer prevention and therapy.

## 1. Introduction

Lung cancer is a leading cause of death in the world. Non-small-cell lung cancer (NSCLC) accounts for nearly 80% of cases and has 13% overall 5-year survival rate [1]. The primary limitation in the effective treatment of NSCLC is an incomplete understanding of its specific cellular and molecular pathogenic elements. Accumulating evidence has shown that inflammation plays an important role in lung cancer development, especially those induced by cigarette smoke and other noxious particles and gases [2, 3]. The interactions between tumor cells with the microenvironment are involved in the pathogenesis of lung cancer.

CD4+T helper cells play a key role in the pathogenesis of inflammatory and autoimmune diseases via the production of distinctive sets of cytokines [4, 5]. CD4+T helper cells were originally classified into Th1 and Th2 subsets based on the secretion of cytokines [6]. Traditionally, research in lung cancer immunity has focused almost exclusively on Th1/Th2 cell balance [7]. Recently, various studies have identified a new lineage of T helper cells, designated Th17, and have established the pivotal role of this cell subset in the pathogenesis of inflammatory conditions and cancers [8]. Th17 cells are characterized by the synthesis of IL-17A, IL-17F, IL-21, IL-22, and IL-26 and express the key transcription factor: retinoic acid receptor-related orphan receptor *γ*t (ROR*γ*t) [9]. Transforming growth factor-*β* (TGF-*β*), IL-1, and IL-6 or IL-21 are shown to induce the differentiation of Th17 cells [9]. Until now, information on Th17 cells and related cytokines in lung cancer hosts is still limited. In particular, the role of Th17 cells and IL-17 in lung cancer immunity has been controversial. IL-17 promotes angiogenesis in tumor models and IL-17 expression correlates well with the TNM stage in human NSCLC [10, 11]. In contrast, antitumor functions of IL-17 have also been noted. For instance, IL-17 may enhance IFN-g-producing tumor-infiltrating NK and T cells to inhibit lung tumor metastasis [12, 13].

The interleukin-27 (IL-27) was identified in 2002 and belongs to the IL-6/IL-12 family. It includes Epstein-Barr virus-induced gene 3 (EBI3) and IL-27p28 and is mainly produced by activated antigen-presenting cells, such as dendritic cells (DCs), monocytes, and macrophages, mediating its effects by interaction with distinct IL-27 receptor subunits [14, 15]. It is now appreciated to be an important regulator of innate and adaptive immunity and has broad inhibitory effects on DCs, Th17 cells, and Th1 cells [16–18]. On the other hand, IL-27 exerts potent antitumor effects against various tumor models via different mechanisms, depending on the characteristic of each tumor [19, 20]. However, still little is known about IL-27 alterations associated with NSCLC. Furthermore, little information is available on the association between IL-27 expression and Th17 cells in NSCLC patients.

In the present study, we hypothesized that Th17 and IL-27 are involved in the inflammatory response in NSCLC. We investigated the proportion of Th17 cells in peripheral blood mononuclear cells (PBMCs) of NSCLC patients and healthy volunteers by flow cytometry and tested the concentrations of serum inflammatory mediators such as IL-27 and IL-17 by enzyme-linked immunosorbent assay (ELISA). In addition, we examined the mRNA expression of IL-27, IL-27, and ROR*γ*t in the peripheral blood by real-time quantitative polymerase chain reaction (QPCR). Finally, we analysed the correlation between IL-27 expression and Th17 cells.

## 2. Materials and Methods

### 2.1. Study Subjects

Nineteen NSCLC patients (male 13, female 6, age range: 37–68 yrs) and nineteen healthy volunteers (male 12, female 7, age range: 41–65 yrs) from December 2013 to October 2014 were enrolled in this study. The diagnosis of lung cancer was established by demonstration of malignant cells on biopsy specimen. None of the patients had received radiotherapy, chemotherapy, or other medical interventions. Healthy controls with a history of malignancies or autoimmune disorders were excluded from this study. This study was approved by the Medical Ethical Committee of the First People's Hospital of Nanning (Nanning, China) and the Medical Ethical Committee of the Eighth People's Hospital of Nanning (Nanning, China), and written informed consent was obtained from all participants.

### 2.2. Sample Collection and Processing

Thirty-milliliter venous blood samples were collected by venipuncture from all the participants and were divided into 3 parts. All samples were taken in a blinded manner for clinical information. Approximately ten milliliters of venous blood was removed erythrocytes with RBC lysis buffer (Sigma-Aldrich) for 10 minutes at room temperature and the remaining cells were washed twice with cold PBS and centrifuged at 1200 rpm for 10 minutes. Fresh peripheral-blood mononuclear cells (PBMCs) were used for intracellular cytokine staining within 1 h. Approximately ten milliliters of venous blood was separated serum for real-time quantitative polymerase chain reaction (QPCR). The rest was separated serum and serum was frozen at −80°C immediately after centrifugation for later determining of concentrations of cytokines by enzyme-linked immunosorbent assay analysis (ELISA).

### 2.3. Immunofluorescence Labeling and Flow Cytometry

The expression marker on T cells was determined by flow cytometry after surface staining or intracellular staining with phycoerythrin cyanine-5-conjugated anti-human CD4 (PE-Cy5-CD4) and phycoerythrin-conjugated anti-human IL-17 (PE-IL-17). These human Abs were purchased from BD Biosciences (San Diego, CA). Appropriate isotype controls were performed for each experiment. Briefly, PBMCs were stimulated with phorbol myristate acetate (PMA, 25 ng/mL, Sigma-Aldrich, USA) and ionomycin (1 *μ*g/mL, Sigma-Aldrich, USA) in the presence of GolgiStop (BD Biosciences) for 4 h. The cells were washed and then fixed/permeabilized in the eBioscience fixation/permeabilization and permeabilization buffers, stained with fluorescent antibodies against CD4 and IL-17. Flow cytometry was performed on a BD FACS Calibur flow cytometer and analyzed by using FCS ExpressV4 software.

### 2.4. Real-Time Quantitative PCR

For quantitative real-time polymerase chain reaction (PCR) analysis of mRNA expression of the ROR*γ*t in peripheral blood, total RNA was extracted from samples with TRIzol (Invitrogen, Life Technologies) according to the manufacturer's instructions. cDNA was prepared using oligo(dT) primers (RevertAid First Strand cDNA Synthesis Kit, Fermenta). Quantitative RT PCR was performed in duplicate with SYBR Green I using a LightCycler (iCycler IQ, BioRad, USA) according to the manufacturer's instructions and using the following primers: IL-27: 5′-TGCCAGGAGTGAACCTGTACC-3′ and 5′-CGTGGTGGAGATGAAGCAGA-3′; IL-17: 5′-GGAATCTCCACCGCAATGAG-3′ and 5′-ACACCAGTATCTTCTCCAGGC-3′; ROR*γ*t: 5′-GCCAGAATGACCAGATTGTGCTT-3′ and 5′-AAGGCACTTAGGGAGTGGGAGA-3′; and *β*-actin: 5′-ACACTGTGCCCATCTACG-3′ and 5′-TGTCACGCACGATTTCC-3′. The identity of the amplified products was examined using 12% polyacrylamide gel electrophoresis and melt curve analysis, and the ratios of each gene product to *β*-actin product were used as indices of IL-27, IL-17, and ROR*γ*t mRNA expression.

### 2.5. Measurement of Cytokines in Serum

The concentrations of IL-17 and IL-27 in serum were measured by ELISA kits according to the manufacturer's protocols (all kits were purchased from R&D Systems, Minneapolis, MN). All samples were assayed in duplicate.

### 2.6. Statistical Analysis

Quantitative data were expressed as mean ± standard deviation. The nonparametric Mann-Whitney *U* tests were used to compare data between NSCLC patients and healthy controls. A value of *P* < 0.05 was considered statistically significant. Correlation analysis was performed using Spearman's rank correlation coefficient. All statistical analyses were performed by using SPSS statistical software version 16 (SPSS Inc., Chicago, IL).

## 3. Results

### 3.1. Expression and Correlation Analysis of IL-27 and IL-17 Levels in Serum of NSCLC Patients

**Figure 1:**
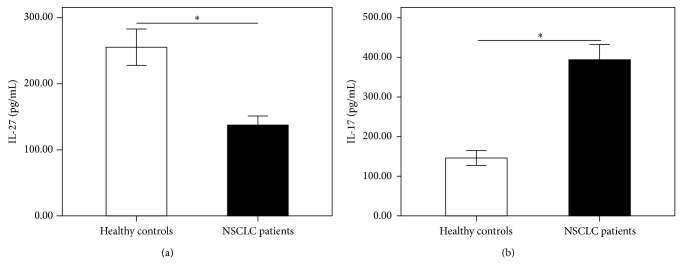
The levels of IL-27 and IL-17 were measured by using ELISA. Serum IL-27 levels (a) were significantly decreased in NSCLC patients as compared to healthy controls. IL-17 levels (b) in serum were significantly higher in NSCLC patients than in healthy controls. Results are expressed as pg/mL (mean ± SD). *n* = 19; ^∗^ *P* < 0.001.

IL-27 and IL-17 levels in serum samples from NSCLC patients and normal controls were assessed by ELISA. Serum IL-27 levels were significantly decreased in NSCLC patients (241.74 ± 38.13 pg/mL) as compared to normal controls (318.13 ± 16.38 pg/mL, *P* < 0.0001) (Figure 1(a)). IL-17 levels in serum were significantly higher in NSCLC patients (394.17 ± 38.29 pg/mL) than in healthy controls (147.27 ± 20.66 pg/mL, *P* < 0.0001) (Figure 1(b)). Additionally, the expression of IL-27 in the serum of NSCLC patients showed no correlation with that of IL-17 (*r* = −0.211, *P* = 0.387).

### 3.2. Expression and Correlation Analysis of IL-27 and IL-17 mRNA Expression in Peripheral Blood of NSCLC Patients

**Figure 2:**
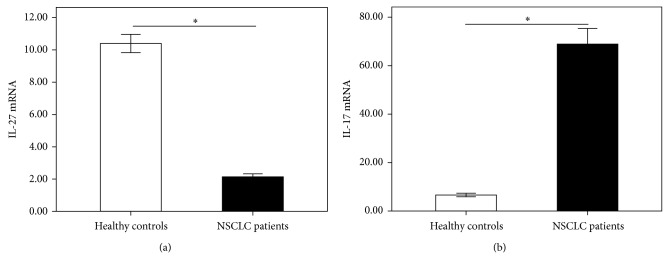
The mRNA expressions of IL-27 and IL-17 in peripheral blood were measured by using QPCR. The mRNA levels of IL-27 (a) were significantly lower in NSCLC patients than in healthy controls. IL-17 mRNA levels (b) were significantly higher in NSCLC patients than in healthy controls. Results are expressed as pg/mL (mean ± SD). *n* = 19; ^∗^ *P* < 0.001.

As depicted in Figure 2, the mRNA levels of IL-27 in peripheral blood of NSCLC patients (2.14 ± 0.19) were significantly lower than those in healthy individuals (10.40 ± 0.57, *P* < 0.0001) (Figure 2(a)). The mRNA levels of IL-17 in peripheral blood were significantly higher in NSCLC patients (68.98 ± 6.66) than in healthy controls (6.59 ± 0.73, *P* < 0.0001) (Figure 2(b)). Additionally, the mRNA levels of IL-27 in peripheral blood of NSCLC patients showed no correlation with that of IL-17 (*r* = −0.439, *P* = 0.06).

### 3.3. CD4^+^IL-17^+^ (Th17) Cells Proportion and ROR*γ*t mRNA Expression in Peripheral Blood of NSCLC Patients

**Figure 3:**
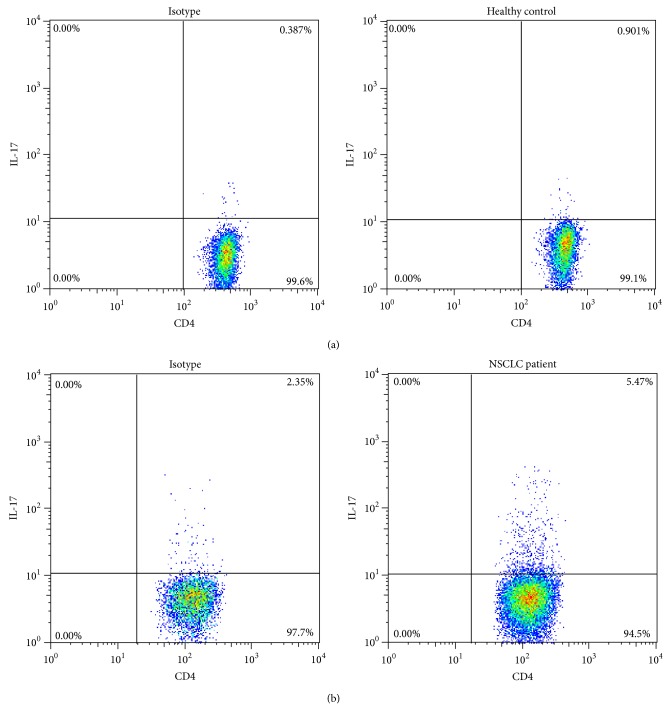
The frequency of CD4+IL-17+ (Th17) cells in peripheral blood was measured by flow cytometry. (a) Representative FACS staining for IL-17A in gated CD4+ T cells from a single patient in healthy controls. (b) Representative FACS staining for IL-17A in gated CD4+ T cells from a single patient in NSCLC patients.

**Figure 4:**
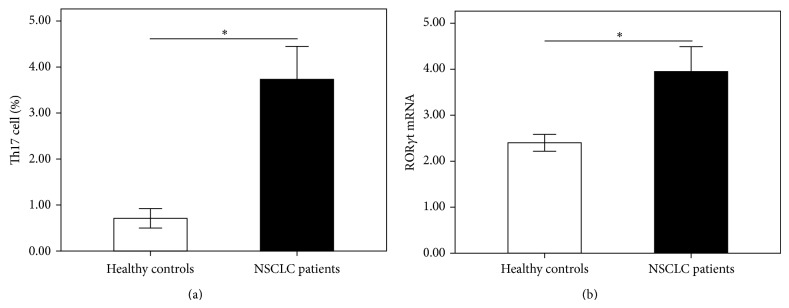
The frequency of CD4+IL-17+ Th17 cells was measured by using flow cytometry, and the mRNA expression of ROR*γ*t in peripheral blood was measured by using QPCR. The proportion of Th17 cells (a) was significantly increased in NSCLC patients compared with healthy controls. The mRNA expression of ROR*γ*t (b) was significantly higher in NSCLC patients than in healthy controls. Results are expressed as pg/mL (mean ± SD). *n* = 19; ^∗^ *P* < 0.001.

**Figure 5:**
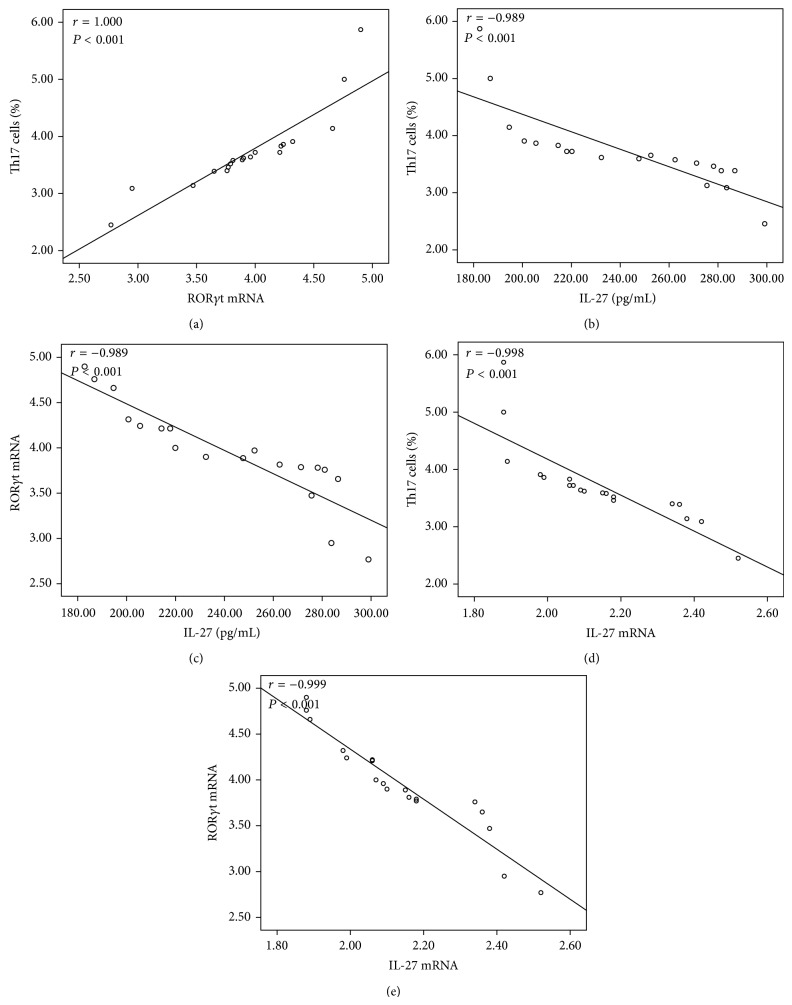
Correlations between (a) the frequency of Th17 cells and ROR*γ*t mRNA, (b) the frequency of Th17 cells and IL-27 levels, (c) ROR*γ*t mRNA and IL-27 levels, (d) the frequency of Th17 cells and IL-27 mRNA, and (e) ROR*γ*t mRNA and IL-27 mRNA in NSCLC patients. Data were determined by Spearman's rank correlation coefficients.

We then performed flow cytometry on PBMCs from NSCLC patients and healthy controls to identify Th17 cells. Representative flow cytometry results were shown in Figure 3 and the quantitative results were shown in Figure 4(a). The proportion of Th17 cells in the PBMCs was significantly increased in NSCLC patients (0.71 ± 0.21) compared with healthy controls (3.73 ± 0.72) (*P* < 0.001, Figure 4(a)). ROR*γ*t was described as important transcription factor involved in the development and function of Th17 cells. Since the proportion of Th17 cells in the peripheral blood from NSCLC patients increased, we investigated the mRNA expression of ROR*γ*t by RT-QPCR. As expected, we found a significantly level of ROR*γ*t mRNA in NSCLC patients (3.95 ± 0.54) compared to healthy controls (2.40 ± 0.18) (*P* < 0.001, Figure 4(b)). In addition, we noted that the level of ROR*γ*t mRNA in the peripheral blood of NSCLC patients was significantly and positively correlated with Th17 cells numbers (*r* = 1.000, *P* = 0.001) (Figure 5(a)). These results indicated that activated Th17 cells are likely to play a pathogenic role in the development of NSCLC.

### 3.4. Correlation Analysis of IL-27 and Th17 Cells and ROR*γ*t mRNA in the Peripheral Blood of NSCLC Patients

The protein levels of IL-27 were significant negatively correlated with the frequencies of Th17 cells and with the ROR*γ*t mRNA in the peripheral blood of NSCLC patients (*r* = −0.989, *P* < 0.001; *r* = −0.989, *P* < 0.001, Figures 5(b) and 5(c)). Similarly, the mRNA levels of IL-27 were also significantly and negatively correlated with the frequencies of Th17 cells (*r* = −0.998, *P* < 0.001, Figure 5(d)) and with the ROR*γ*t mRNA (*r* = −0.999, *P* < 0.001, Figure 5(e)).

## 4. Discussion

Lung cancer is considered a T-cell-mediated inflammatory disease, but its etiology and pathology have not been elucidated. In this study, we explored the involvement of IL-27 and Th17 cells in the pathogenesis of NSCLC, given that IL-27 has a close relationship with T helper subsets and plays a potential role in immunity and antitumor function [21–23]. In this study we found that IL-27 levels were decreased in the serum samples of NSCLC patients as compared with normal controls. In addition, the frequencies of Th17 cells were increased in NSCLC patients, accompanied by the upregulation of IL-17 and ROR*γ*t. Furthermore, IL-27 negatively correlated with the numbers of Th17 cells and the ROR*γ*t mRNA. Taken together, these results indicate that IL-27 production was negatively correlated with the commitment of Th17 cells and might have a therapeutic potential in lung cancer.

Th17 cells are now defined as a separate CD4+ T-cell subset distinct from the Th1 and Th2 cells, driving inflammatory responses in several T-cell driven autoimmune diseases [24–26]. More recently, multiple lines of evidence have indicated that Th17 cells are involved in lung cancer [27–29]. A higher level of IL-17A mRNA and protein expression was noted in lung CD4+ T cells from NSCLC patients as compared to healthy controls [30, 31]. Functionally, overexpression of IL-17 in tumor cell lines promotes angiogenesis and tumor growth when the tumors are implanted in immune-compromised mice [32]. These reports indicate that IL-17-mediated responses promote tumor development. In contrast, recent reports indicate that tumor growth in subcutaneous tissue and lung tumor metastasis are enhanced in IL-17−/− mice [12, 13]. It implicates that IL-17-mediated responses are protective against tumor development. In this study, we analyzed the proportion of Th17 cells and the expression of IL-17 and ROR*γ*t2 in NSCLC patients by flow cytometry, real-time quantitative PCR, and ELISA. Consistent with Li et al. [29], the results showed that the Th17 subset was increased in NSCLC and was accompanied by the upregulation of both the mRNA and protein level of IL-17 as well as the Th17-specific transcription factor ROR*γ*t. Thus, these results indicate that the inflammatory function of Th17 may play important roles in the pathogenesis of lung cancer.

IL-27, mainly produced by activated antigen-presenting cells, was shown to play key roles in regulating T-cell functions [18]. It has been reported that IL-27 can effectively inhibit the expression of Th17-specific transcription factor ROR*γ*-t through STAT1-dependent pathway [21] and can play a negative regulatory role in the Th17-type immune response by inhibiting the proliferation of mature polarized CD4+T cells [33]. It can also inhibit IL-17-polarizing cytokines from DCs, which in turn decreases IL-17 secretion from T cells [34]. These results indicated that IL-27 could antagonize the function of IL-17. Considering the fact [17] that IL-27 is correlated with Th17 cell, the levels of IL-27 in NSCLC patients were also evaluated. In the present study, a significant decreased expression of IL-27 protein and mRNA in peripheral blood was observed in NSCLC patients. In addition, IL-27 and Th17 cell percentage and ROR*γ*t mRNA was negatively correlated, suggesting that the inhibition function may decline due to the decrease of IL-27 level in peripheral blood of NSCLC patients, resulting in increased differentiation activation of Th17 cells. Therefore, IL-27 may play an anti-inflammatory role in the pathogenesis of cancer-associated immunological abnormalities. It has been demonstrated that IL-27 mediated inhibition of angiogenesis in various malignancies [3, 5]. Kachroo et al. [35] reported that IL-27 can promote epithelial-mesenchymal transition (EMT) and inhibit cell migration and the production of angiogenic factors in human NSCLC through STAT1-dependent pathway. IL-27 can also downregulate proangiogenic molecules including VEGF, angiopoietins, and matrix metalloproteinases (MMP) and upregulate the antiangiogenic chemokines including CXCL9 and CXCL10 in vitro [36, 37]. In addition, IL-27 directly suppresses tumorigenicity through downregulation of vimentin, COX-2, and PGE2 on lung cancer cells [38]. Although IL-27 is negatively correlated with Th17 cell, our data showed that no association of IL-27 with IL-17 was found in NSCLC patients, indicating that the higher circulating IL-17 levels may originate from Th17 cells and any other NK cells and that IL-27 levels may have little effect on the secretion of IL-17 in NSCLC patients. Thus, the effects of IL-27 on the secretion of IL-17 should be further studied in the future.

In summary, we found that IL-27 levels were significantly decreased in the serum samples of NSCLC patients. The frequencies of Th17 cells were significantly elevated increased in NSCLC patients, accompanied by the upregulation of IL-17 and ROR*γ*t. In addition, IL-27 negatively correlated with the numbers of Th17 cells and the ROR*γ*t mRNA. These findings suggest that Th17 cell may play an important role in lung cancer and suggest that better understanding of the regulatory effects of IL-27 on Th17 cells may shed light on potential new targets in cancer prevention and therapy.

## Highlights

1. Expression of IL-27 was lower in NSCLC patients compared with normal controls.
2. The frequency of Th17 cells was increased in NSCLC patients, accompanied by the upregulation of IL-17 and ROR*γ*t.
3. IL-27 negatively correlated with the number of Th17 cells and the ROR*γ*t mRNA.
4. IL-27 might inhibit Th17 differentiation in NSCLC patients and might have a therapeutic potential in lung cancer.
